# Advancements in Artificial Intelligence-Based Diagnostic Tools Used to Detect Fungal Infections: A Systematic Review

**DOI:** 10.3390/diagnostics16030450

**Published:** 2026-02-01

**Authors:** Noir M. Albuqami, Lina M. Alkahtani, Yara A. Alharbi, Duaa A. Aljuhaymi, Ragheed D. Alnufaei, Alaa A. Al Mashaikhi, Anwar A. Sayed

**Affiliations:** 1 College of Nursing, King Saud bin Abdulaziz University for Health Sciences, Jeddah 21423, Saudi Arabia; 2 College of Medicine, Taibah University, Madinah 42353, Saudi Arabia; 3 College of Medicine, Princess Nourah bint Abdulrahman University, Riyadh 11671, Saudi Arabia; 4 Department of Medicine, King Abdulaziz University Hospital, Jeddah 21589, Saudi Arabia; 5 Department of Medicine, King Faisal Medical Complex, Taif 21944, Saudi Arabia; 6 Department of Basic Medical Sciences, College of Medicine, Taibah University, Madinah 42353, Saudi Arabia

**Keywords:** artificial intelligence, diagnostics, fungal infection, systematic review

## Abstract

**Background:** Fungal infections are considered a global health concern, resulting in high morbidity and mortality rates, especially in immunocompromised individuals. Traditional diagnostic techniques, such as microscopy, culture, and polymerase chain reaction (PCR), suffer from low sensitivity, long processing time, and accessibility challenges, especially in resource-limited settings. Artificial intelligence (AI) and machine learning (ML) tools have demonstrated potential to enhance diagnostic accuracy and efficiency. This systematic study assesses the progress, precision, and efficacy of AI-driven diagnostic tools for fungal infections within various clinical contexts in comparison to traditional procedures. **Methods:** A systematic review was conducted according to PRISMA principles. Literature searches were conducted in PubMed, ScienceDirect, Web of Science, and Ovid, focusing on research employing AI models to diagnose fungal infections. The inclusion criteria were research that compared AI-based tools with conventional diagnostic methods in terms of sensitivity, specificity, and accuracy. Data extraction and quality evaluation were performed utilizing validated instruments, such as the Methodological Index for Non-Randomized Studies (MINORS). **Results:** Eleven research studies met the inclusion criteria: six retrospective and five prospective investigations. AI models, such as convolutional neural networks (CNNs), Faster R-CNN, VGG19, and MobileNet, have improved diagnostic accuracy, sensitivity, and specificity compared to traditional methods. However, differences in dataset quality, model validation, and real-world applicability remain as limitations. **Conclusions:** AI-driven diagnostic technologies provide significant benefits in identifying fungal infections, improving the speed and accuracy of diagnoses. However, additional extensive investigations and clinical validation are required to improve model generalizability and facilitate smooth incorporation into healthcare systems.

## 1. Introduction

Fungal infections cause more than 1.6 million deaths annually and affect more than 1 billion individuals worldwide; therefore, they are a global health concern. Their burden is comparable to that of tuberculosis and exceeds that of malaria [1]. According to Hsu and Lee, fungal infections are typically classified based on the depth of tissue involvement. Superficial infections such as dermatophytosis and cutaneous candidiasis are limited to the epidermis and associated skin structures, whereas subcutaneous mycoses extend to the dermis and subcutaneous tissue; in contrast, invasive fungal infections systemically affect the internal organs and may present with secondary cutaneous manifestations [2].

Osman et al. highlighted the increasing public health threat of invasive fungal infections in the Middle East and North Africa (MENA) region, particularly among individuals who are immunocompromised and patients in the intensive care unit [3]. However, epidemiological data are limited and conflicting. Candidemia, which is the most frequently reported invasive fungal infection, has high incidence and mortality rates (10.5–58%) in countries such as Saudi Arabia, Qatar, and Egypt; furthermore, invasive aspergillosis is also common, especially among patients with hematologic malignancies and those who have received transplants, and its prevalence is notably high in Egypt and Iran [2]. Other fungal diseases, including cryptococcal meningitis, Pneumocystis pneumonia, and mucormycosis, have been underreported; however, they are associated with high mortality rates [3]. Gnat et al. identified Aspergillus, Candida, and Cryptococcus as primary fungal pathogens that often affect patients who are immunocompromised [4]. Major risk factors for fungal infections include chemotherapy, organ transplantation, prolonged antibiotic use, and conditions such as human immunodeficiency virus/acquired immune deficiency syndrome, cancer, and diabetes. Additionally, environmental factors, such as global warming, contribute to widespread infections.

Despite the increasing incidence and severity of such infections, their diagnoses remain challenging because of the inherent limitations of conventional diagnostic methods [4]. According to Fang et al., fungal infections are typically diagnosed using microscopy, cultures, and histopathology; however, these methods have low sensitivity, provide delayed results, and are invasive, resulting in diagnostic delays and increased mortality [5]. Although molecular techniques, such as PCR, provide improved speed and accuracy, they are costly and inaccessible in many low-income and middle-income areas. Alternative nonculture-based methods, such as antigen detection assays (1,3)-β-D-glucan or galactomannan, are faster and less invasive options, but they may yield false-positive results or have reduced sensitivity in certain populations. Additionally, lateral flow devices and biosensors may improve diagnostic access in resource-limited environments [5].

Ahmad et al. reported that the role of artificial intelligence (AI) in medicine is rapidly developing and has the potential to transform patient care by advancing personalized treatment approaches [6]. AI can be used to modify medical interventions to satisfy the needs of patients. Machine learning (ML) is a branch of AI that involves computational systems that utilize layered algorithms to analyze large and complex datasets and uncover patterns and insights that aid clinical decision-making, which can improve clinical diagnoses [6]. Therefore, the utility of AI in various areas of medicine, particularly for enhancing diagnostic accuracy and clinical decision-making, is increasing. Nakagawa et al. developed a deep learning (DL)-based AI system that exhibited favorable performance when used to evaluate superficial esophageal squamous cell carcinoma with depths that required invasive diagnostic methods [7]. An accurate assessment of the tumor invasion depth is essential because it plays a pivotal role in determining the most appropriate treatment strategy [7]. Zhang et al. demonstrated that ML improved early and accurate detection of bacterial infections by analyzing clinical data, laboratory results, and genetic information; therefore, AI has been effectively applied for conditions such as sepsis and respiratory infections to identify diagnostic patterns that are often missed when using conventional methods [8]. Yuan et al. developed an AI model using the eXTreme gradient boosting (XGBoost) algorithm trained on intensive care unit electronic health records and achieved an accuracy rate of more than 80%; furthermore, the sensitivity and specificity of this model were better than those of the Sequential Organ Failure Assessment score because it continuously analyzed real-time clinical data, including vital signs and laboratory results, without disrupting the clinical workflow, while enhancing diagnostic accuracy [9]. AI has effectively detected sepsis and bacterial infections by using data from electronic health records; however, its application for fungal diagnostics remains limited. Because previous research on fungal diagnostics has revealed a critical gap comprising poor clinical validation, short datasets, and a narrow emphasis on fungal species, the underutilization of AI in this field has gained attention. Therefore, this systematic review aimed to systematically review and synthesize evidence on the diagnostic performance and clinical relevance of AI-based tools in detecting fungal infections, in the context of heterogeneous clinical conditions, addressing existing gaps and proposing directions for future research.

## 2. Materials and Methods

### 2.1. Review Design

This systematic review was conducted in accordance with the Preferred Reporting Items of Systematic Reviews (PRISMA) guidelines to ensure minimal bias in study selection (File S1). The study protocol was registered a priori with PROSPERO (CRD42024619348). Ethical approval was not required due to the nature of this study [10,11].

### 2.2. Research Question

In patients suspected of fungal infections (P), how accurately do AI-based diagnostic tools (I) detect the infection compared with conventional diagnostic methods such as culture, microscopy, or expert interpretation (C), as measured by sensitivity, specificity, and accuracy (O)?

### 2.3. Eligibility Criteria

Studies were selected based on predefined inclusion and exclusion criteria:

Inclusion Criteria:

- Included patients with suspected or confirmed fungal infections in clinical settings
- Assessed AI-based diagnostic tools, including machine learning, deep learning, image analysis, or biomarker detection
- Compared AI tools with standard diagnostic methods such as microscopy, cultures, or polymerase chain reaction
- Reported diagnostic accuracy metrics, including sensitivity and specificity
- Study designs included randomized controlled trials, cross-sectional studies, or cohort studies with comparative data

Exclusion Criteria:

- Nonhuman or nonclinical studies
- Studies focusing on non-AI diagnostic methods
- Absence of diagnostic accuracy metrics
- Lack of comparative analysis
- Non-original studies, including reviews, editorials, or case reports

#### 2.3.1. Population

Patients with suspected or confirmed fungal infections in clinical settings.

#### 2.3.2. Index Test

Artificial intelligence-based diagnostic tools, including machine learning, deep learning, digital and automated image analysis, biomarker detection, and prediction models, were evaluated and compared with standard diagnostic methods for fungal infections

#### 2.3.3. Reference Standard

Standard diagnostic methods, including microscopy, fungal culture, and polymerase chain reaction, were used as reference standards. AI-based diagnostic tools were compared directly with these methods to assess diagnostic accuracy.

### 2.4. Outcomes

Diagnostic accuracy was evaluated using sensitivity and specificity. Comparisons between AI-based tools and standard diagnostic methods were performed to determine relative diagnostic performance.

### 2.5. Eligible Studies

Eligible studies included randomized controlled trials, cross-sectional studies, and cohort studies with comparative diagnostic data.

### 2.6. Information Sources and Search Strategy

In November 2024, a systematic search was conducted in the following databases: PubMed, Ovid, ScienceDirect, Web of Science. Additional articles were identified through manual screening of reference lists of the included studies. The search strategy used the following keywords in all databases: (Fungal Infections OR Mycoses OR Aspergillosis OR Candidiasis OR Cryptococcosis) AND (Artificial Intelligence OR Machine Learning OR Deep Learning OR Neural Network) AND (Diagnosis OR Diagnostic Imaging OR Sensitivity OR Specificity)

### 2.7. Study Selection and Screening

All identified articles were imported into the Rayyan systematic review platform. Title and abstract screening were independently performed by three reviewers. Full-text screening was subsequently conducted by two authors, and any discrepancies were resolved by a third reviewer [12].

### 2.8. Data Extraction

Extracted data included:

- Study characteristics (journal name, study design, year, country, and sample size)
- Participant demographics (age, sex, comorbidities, and smoking status)
- Intervention details (type of AI tool, comparator, duration, outcomes, measurement tools, and timing of outcome assessment)

### 2.9. Risk of Bias and Quality Assessment

The methodological quality and risk of bias of the included studies were assessed using the Methodological Index for Nonrandomized Studies (MINORS) tool for nonrandomized studies (File S2). This validated tool evaluates key methodological aspects such as clarity of the study aim, unbiased outcome assessment, and data collection methods. Each item was scored from 0 (not reported) to 2 (adequate), with higher scores indicating better methodological quality. Two authors independently conducted the assessments, and a third author cross-checked the results to ensure accuracy [13].

## 3. Results

**Figure 1:**
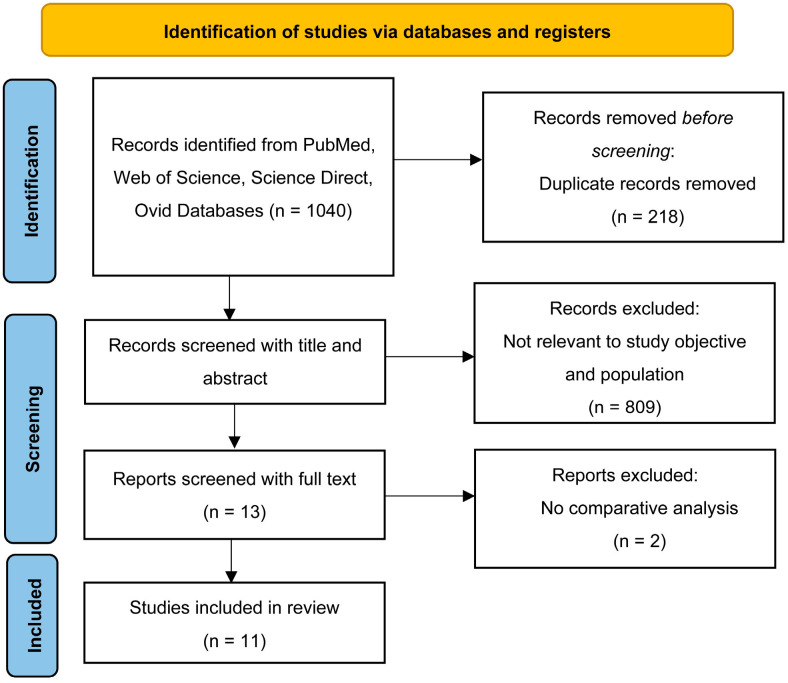
PRISMA flow diagram illustrating the systematic screening, eligibility assessment, and inclusion of studies in the review.

The included studies were mostly imaging-based; however, few implemented multimodal approaches that combined the use of clinical or laboratory information with imaging, as well as non-imaging molecular and spectroscopic inputs. Our systematic search yielded 1040 publications; of these, 248 were from PubMed, 191 were from Web of Science, 525 were from ScienceDirect, and 76 were from Ovid. After the removal of duplicate articles, 822 articles remained. After preliminary application of the inclusion and exclusion criteria, 13 articles were retained for further analysis. Finally, only 11 articles published between June 2020 and January 2023 were included because two did not include a comparative analysis (Figure 1).

This review included 11 studies (six retrospective and five prospective studies) that provided a comprehensive overview through diverse study designs. Geographically, five studies were performed in China (including one performed in Guangxi Zhuang), two were performed in Iran, one was performed in Egypt, one was performed in South Korea, and one was performed in Italy. One study included data from multiple countries.

These studies used advanced AI models, such as convolutional neural networks (CNNs), Faster R-CNN, VGG19, and MobileNet. The sample sizes varied from large patient datasets to small, focused image analyses. Applications ranged from pneumonia and retinal diseases to tumor detection and infectious diseases, thus highlighting the broad clinical relevance of AI. Table 1 summarizes the characteristics of the included studies.

### 3.1. Analysis of Bias, Quality Assessment, and Determination of the Level of Evidence

The methodological quality of the nonrandomized studies included in this review was assessed using the MINORS tool. The total scores of the studies ranged from 8 to 17; the maximum possible scores of noncomparative and comparative studies were 16 and 24, respectively. Arem Lim et al. emphasized that studies with higher scores demonstrated better methodological quality and a lower risk of bias [13]. For example, the study by Kim et al. had a score of 17, indicating strong methodological rigor with adequate statistical analyses and appropriate follow-up periods [16]. In contrast, studies such as that by Wei et al. and Li et al. had a score of 8, primarily because of the absence of prospective data collection and inadequate endpoint assessment [17,22]. Common methodological limitations included the absence of prospective sample size calculations and unbiased endpoint assessments. Overall, the study quality varied, and several studies exhibited a moderate to high risk of bias. These issues underscore the need for cautious interpretation because design flaws may weaken the validity of the findings.

### 3.2. Respiratory System

AI-based diagnostic models, including both imaging-based and multimodal approaches (Implementing clinical and laboratory data with CT chest features), models exhibited good performance when used to detect pulmonary fungal infections [17,21]. Li et al. reported that ML models that used routine clinical indicators such as serum β-D-glucan and chest computed tomography (CT) features exhibited areas under the receiver-operating characteristic curve (AUCs) higher than 0.90; additionally, both sensitivity and specificity for *Pneumocystis jirovecii* pneumonia were more than 80%. Wang et al. developed IPA-NET, which is an AI-based DL diagnostic model, to allow early detection of invasive pulmonary aspergillosis using CT and found that it achieved accuracies of 96.8% and 89.7% during internal testing and external testing, respectively, thus exceeding the accuracy of conventional methods. These findings imply that the accuracy of identifying fungal infections of the lung can be increased by combining imaging with clinical indicators.

### 3.3. Dermatological System

AI in the field of dermatology has mainly focused on onychomycosis and other fungal skin lesions [16,22,24]. Zhu et al. demonstrated a multistage Faster R-CNN ensemble with 95.7% accuracy for classifying nail disorders and 87.5% accuracy for detecting onychomycosis. Kim et al. reported that CNN models performed approximately as well as dermatologists who used dermoscopy; specifically, the CNN models exhibited 71.5% accuracy and sensitivity and specificity similar to those of the dermatologists. Wei et al. found that DenseNet201 had the highest diagnostic accuracy for skin lesions (87.9%). These studies showed that AI can non-invasively enable early detection of fungal skin diseases.

### 3.4. Ocular System

AI tools have been used to study ophthalmic fungal infections, especially keratitis [15,19,20]. Soleimani et al. reported that AI had 99.3% accuracy when used to differentiate healthy corneas from those with keratitis using slit-lamp images; however, the accuracy decreased to 84% when distinguishing fungal keratitis from bacterial keratitis. Tang et al. reported that using transfer learning with in vivo confocal microscopy images to detect *Fusarium* and *Aspergillus* species resulted in accuracy rates of more than 81% and 75%, respectively, thus indicating that AI can help identify species. Essalat et al. tested several CNN models using these images and found that DenseNet161 exhibited the best results (96.9% accuracy and 97.8% specificity). These results indicate that combining ocular imaging with DL can result in a quick and accurate diagnosis of fungal keratitis and may reduce misdiagnoses in clinics.

### 3.5. Otolaryngology

Mao et al. demonstrated that ensemble CNN models, such as ResNet101, SENet101 and EfficientNetB6, using otoendoscopic images, achieved superior diagnostic performance compared with that of single architectures for otomycosis. Notably, the ensemble classifier exhibited 92.4% accuracy, and sensitivity and specificity higher than 94%, thus establishing DL models as a valuable adjunct for fungal infections of the ear, nose, and throat [18].

### 3.6. Non-Imaging and Molecular AI Diagnostic Approaches

Several studies have also integrated spectral and biochemical data [14,23]. Xu et al. combined single-cell Raman spectroscopy with ML to rapidly identify clinical fungal infections with support vector machines and achieved 98% accuracy for classifying fungal species. Elkadi et al. applied Fourier-transform infrared spectroscopy with a partial least-squares discriminant analysis and obtained accuracy higher than 90% and specificity up to 100% after data augmentation. These results suggest that AI can effectively process molecular-level data and is a non-invasive and rapid alternative to traditional fungal culture methods.

### 3.7. Complications

Details regarding complications, such as those associated with diagnostic delays or model inaccuracies, have not been systematically reported. Although studies have aimed to address the limitations of conventional diagnostic methods, potential adverse outcomes that could occur when implementing these AI models in clinical practice should be assessed.

### 3.8. Classification Performance

Clinical outcomes have been extensively reported, and the studies included in this review have demonstrated improved diagnostic capabilities. Retrospective studies have highlighted the superior performance of AI models compared with that of traditional methods. Furthermore, models such as VGG19, MobileNet, and Faster R-CNN have shown improved diagnostic performance metrics compared with traditional methods for conditions such as fungal infections and dermoscopic analyses, primarily in retrospective and internally validated studies. A summary of the findings of the articles included in this review is provided in Table 2: Classification of diagnostic performance metrics.

## 4. Discussion

The application of artificial intelligence (AI) in the diagnosis of fungal infections demonstrates substantial technical promise. The findings of this systematic review indicate that AI-driven methodologies, particularly ensemble models and deep learning (DL) architectures, achieve high diagnostic sensitivity, specificity, and accuracy under experimental and retrospective conditions. In such controlled settings, these models suggest potential technical advantages in diagnostic assessment when compared with conventional diagnostic approaches. Nevertheless, it is essential to distinguish between technical classification performance and clinical efficacy. The evidence synthesized in this review is predominantly derived from retrospective datasets and evaluated using standard classification metrics, including accuracy, sensitivity, and specificity. Although these metrics indicate promising diagnostic capability, there remains a notable lack of evidence from randomized controlled trials (RCTs), prospective pre–post implementation studies, usability assessments, or health economic analyses. Consequently, current evidence is insufficient to determine whether AI-based diagnostic tools for fungal infections improve patient outcomes, reduce diagnostic delays in real-world practice, or provide cost-effective alternatives to conventional laboratory methods.

Claims related to enhanced clinical decision-making should therefore be interpreted with caution. Comparative studies assessing AI models against clinician performance, such as those reported by Kim et al., were conducted primarily under experimental or retrospective conditions and do not constitute direct evidence of real-world clinical effectiveness. As such, any inferred benefit to clinical decision-making remains hypothetical until validated through prospective and interventional study designs [16].

Within the domain of fungal diagnostics, several studies illustrate both the strengths and limitations of AI-based approaches [16,18,19,20]. Tang et al. demonstrated that deep learning classifiers outperformed traditional decision tree models for fungal pathogen identification, while Mao et al. reported that ensemble architectures surpassed conventional microscopy for the diagnosis of otomycosis, emphasizing the potential of convolutional neural network-based approaches in image-driven fungal diagnostics. Kim et al. further reported high classification accuracy for onychomycosis detection using convolutional neural networks; however, Soleimani highlighted persistent challenges in subclassifying fungal keratitis and differentiating filamentous fungi from yeasts, underscoring the influence of disease complexity and dataset characteristics on model performance.

Additional evidence supports the applicability of AI across a broader spectrum of fungal infections [17,21,22]. Li et al. demonstrated robust performance of a random forest model for diagnosing Pneumocystis jirovecii pneumonia, while Wei et al. reported high accuracy using multiple deep learning architectures for classifying cutaneous cryptococcosis and talaromycosis lesions. Wang et al. further demonstrated that deep learning-based models achieved high accuracy for the early detection of invasive pulmonary aspergillosis, supporting the feasibility of AI-assisted diagnostics in invasive fungal diseases.

Beyond the studies included in this review, recent fungal-specific research further contextualizes these findings [20,25,26,27,28,29,30]. Rahman et al. applied deep learning models to classify a wide range of fungal genera from microscopic images, demonstrating the feasibility of broad-spectrum AI-based fungal identification while highlighting challenges related to data imbalance across rare species. A study by Shankarnarayan and Charlebois showed that machine learning approaches have also been evaluated for identifying clinically relevant candida species from microscopy images, with high sensitivity and specificity reported across multiple yeast species. In an ocular mycology study by Tang et al., deep learning classifiers applied to in vivo confocal microscopy images have shown the ability to differentiate pathogenic genera such as Fusarium and Aspergillus, suggesting potential utility in the early and non-invasive diagnosis of fungal keratitis. Additional studies have explored AI-assisted diagnostics for superficial fungal infections using fluorescence microscopy (He et al.), demonstrating the feasibility of automated image analysis for early detection. In pulmonary mycology, investigations into AI-based analysis of computed tomography imaging for chronic pulmonary aspergillosis (CPA Imaging Study) by Angelini and Saha have highlighted both diagnostic potential and the need for disease-specific imaging datasets. Similarly, Tsang et al. demonstrated that AI-based image recognition systems can accurately identify clinically important Aspergillus species, while Pour et al. reported high diagnostic accuracy for superficial fungal infections using convolutional neural network-based systems.

Despite encouraging technical performance, variability in model accuracy across studies remains substantial. This variability appears to be driven primarily by limited dataset size, restricted species diversity, and single-center retrospective study designs rather than inherent limitations of AI architectures. Small and homogeneous datasets increase the risk of overfitting and restrict generalizability, while false-positive results and misclassifications remain clinically relevant concerns. Accordingly, multicenter external validation is essential to assess robustness across diverse patient populations and fungal species.

AI-based diagnostic tools may offer particular advantages in resource-constrained settings, where conventional fungal diagnostics often require specialized laboratory infrastructure and prolonged turnaround times. Deployment via smartphone-based or cloud-enabled platforms may improve diagnostic accessibility; however, model performance remains highly dependent on the quality and diversity of training data. Barriers to adoption, including regulatory constraints, limited clinician trust, and the need for continuous model updates, must be addressed before widespread clinical implementation can be achieved. Overall, the findings suggest that current limitations in AI-based fungal diagnostics are driven primarily by data scarcity, limited species diversity, and retrospective study designs rather than deficiencies in model architecture. Future research should prioritize the development of large, multicenter, species-specific datasets, alongside prospective clinical trials and health economic evaluations, to enable the translation of promising technical performance into clinically reliable diagnostic tools that complement, rather than replace, clinical expertise.

### 4.1. Limitations

Despite the promising results, several critical limitations in the current body of research warrant emphasis. First, the recurring problem of overfitting must be addressed. This occurs when a model essentially memorizes its training data, much like a student who studies only from a specific set of flashcards. It performs flawlessly on that set but struggles with new questions. This issue is often observed in studies with relatively small or uniform datasets, such as those from Wei et al. and Elkadi et al. The high accuracy reported in these settings can be misleading, as it does not guarantee that the model will perform reliably across different patient populations in real-world hospital environments [14,22].

This concern ties directly into a second major limitation: the need for stronger external validation. A model may appear highly effective when tested on data originating from the same institution where it was trained; however, the true test lies in whether its performance generalizes to other settings. While some researchers, such as Wang et al., have taken this crucial step by validating their models using data from separate institutions, many studies have not. Without rigorous multi-center testing, confidence in the real-world clinical performance of these AI tools remains limited [21].

Finally, it is crucial to address the “black box” dilemma. Many of the most powerful AI models, particularly complex deep learning architectures, provide diagnostic outputs without offering clear explanations for their decisions. For clinicians, understanding why a tool suggests a particular diagnosis is non-negotiable for building trust and supporting informed decision-making. Most studies reviewed place substantial emphasis on performance metrics such as accuracy, while devoting considerably less attention to interpretability. Moving forward, it will be essential for researchers to integrate explainable AI (XAI) techniques. Making the reasoning behind an AI model’s output transparent is not merely a technical refinement; it is a fundamental requirement for clinical acceptance and safe implementation.

### 4.2. Recommendations

Across clinical domains, current evidence supports the use of AI primarily as an adjunct screening and triage tool rather than a standalone diagnostic solution for fungal infections. Imaging-based applications in ophthalmology and dermatology show the strongest potential for early detection and referral, while integrated clinical–radiologic models may aid risk stratification in selected respiratory fungal infections, particularly in high-risk populations. Laboratory-based spectroscopy approaches also appear promising for rapid species identification but require further standardization. To enable safe and effective clinical implementation, future AI models should be developed using multi-center datasets with standardized acquisition and labeling, employ patient-level data splits to prevent leakage, undergo rigorous external and prospective validation, and provide calibrated, interpretable outputs that support clinician trust and decision-making.

## 5. Conclusions

This review indicates that AI-based approaches have the potential to support the detection of fungal infections, with the strongest evidence emerging from imaging-driven applications, most notably ophthalmic and dermatologic workflows, as well as from integrated clinical radiologic models in selected respiratory contexts. Across the included studies, CNN-based and transfer-learning strategies frequently achieved high performance on internal datasets; however, results were less consistent for fine-grained tasks, such as differentiating fungal from bacterial infections or performing genus or species-level classification. These tasks often require richer annotations, standardized data acquisition, and greater dataset diversity.

Importantly, the current evidence base is constrained by the risk of overfitting, limited external validation, and insufficient reporting of model interpretability, which collectively limit generalizability and readiness for unsupervised clinical deployment. Future research should prioritize multi-center validation across devices and patient populations, transparent reporting practices, calibration at clinically meaningful thresholds, and the development of interpretable decision-support systems that integrate seamlessly into real-world clinical workflows. Until such evidence is available, AI tools are best positioned as adjuncts for triage and clinical decision support, complementing rather than replacing standard mycological and clinical diagnostic practices.

## Figures and Tables

**Table 1:** Summarizes the characteristics of the included studies.

Author, (Year)	Study Design	Sample Size	System	Data Source	AI Model	Comparator	Outcome	Performance Metric
Elkadi et al., 2021 [14]	Prospective	Training: 16 plasma samples (9 spiked with *Aspergillus*, 7 controls). Oversampling: 200 simulated spectra (100 positive, 100 negative). Testing: 45 mock plasma samples containing drugs and other pathogens as confounders.	Non-imaging and molecular AI diagnostic approaches	Application of Fourier-transform infrared (FTIR) spectroscopy of human plasma samples combined with machine learning (PLS-DA) for the detection of *Aspergillus* species.	Partial Least Squares–Discriminant Analysis (PLS-DA) with variations (BaselineM, AvgSimM, BaselineMs, AvgSimMs).	Standard laboratory testing	Classifying Aspergillus-positive versus Aspergillus-free plasma samples.	Accuracy, sensitivity, and specificity
Essalat et al., 2023 [15]	Retrospective study	4001 IVCM images (1391 AK, 897 FK, 1004 NSK, 743 healthy). Dataset split: 3000 images for training, 1001 images for testing.	Ocular	Development of deep learning models (CNN-based) for automated diagnosis of fungal keratitis (FK), acanthamoeba keratitis (AK), nonspecific keratitis (NSK), and healthy corneas using in vivo confocal microscopy (IVCM) images.	DenseNet161, DenseNet121, ResNet101, ResNet152, VGG19, VGG13.	Clinical diagnosis	Classification of keratitis.	Accuracy, sensitivity, specificity
Kim et al., 2020 [16]	Prospective cohort	90 patients (57 with onychomycosis, 33 with nail dystrophy).	Dermatology	Application of a deep neural network to diagnose onychomycosis from clinical photographs, compared against dermoscopy and dermatologists’ evaluation.	Convolutional Neural Network (CNN)-based deep neural network (ensemble of ResNet-152, VGG-19, and Faster R-CNN for nail plate detection).	Dermatology assessment/dermoscopy.	Diagnosis of onychomycosis.	Sensitivity, specificity and accuracy
Li et al.,2023 [17]	Retrospective	Total: 704 patients with severe pneumonia (256 with PCP, 448 without). Training set: 564 patients. Testing set: 140 patients	Respiratory	Clinical indicators (neutrophil count, globulin, serum β-D-glucan, and chest CT ground-glass opacity).	Logistic Regression, XGBoost, Random Forest (RF) and LightGBM	Conventional clinical diagnosis	Diagnostic identification of PCP.	AUC, sensitivity, specificity, PPV, NPV.
Mao et al., 2022 [18]	Retrospective study	Total dataset: 4000 otoendoscopic images Training set: 2182 images Validation set: 475 images Test set: 120 images	ENT	Use of an ensemble deep learning model combining multiple architectures (ResNet101, SENet101, EfficientNetB6) to classify otomycosis from otoendoscopicimages.	The deep convolutional neural network (CNN) ResNet101, SENet101, EfficientNetB6, Ensemble (set classifier).	Microscopy	Identifying otomycosis from otoendoscopicimages.	Accuracy, specificity and sensitivity
Soleimani et al., 2023 [19]	Retrospective study	Total: 9329 slit-lamp images from 977 patients: Model 1: 2505 healthy + 6824 keratitis images Model 2: 2008 fungal + 4816 bacterial images Model 3: 1643 Aspergillus/Fusarium + 357 Candida images	Ocular	Classification of slit-lamp images into healthy vs. keratitis, bacterial vs. fungal keratitis, and fungal subtypes (filamentous vs. yeast).	Three custom-designed Convolutional Neural Networks (CNNs): Model 1 (Healthy vs. Keratitis), Model 2 (Fungal vs. Bacterial keratitis), Model 3 (Aspergillus/Fusarium vs. Candida).	Microbiological diagnosis	Diagnosis and classification of infectious keratitis.	Accuracy, Sensitivity and specificity
Tang et al., 2023 [20]	Retrospective	3364 IVCM images (from 100 eyes of 100 patients with culture-proven filamentous fungal keratitis).	Ocular	In vivo confocal microscopy images	Inception-ResNetV2 (as feature extractor, with two approaches: 1. Decision Tree classifier (DT model) 2. Deep Learning classifier (DL model, with fully connected layers).	Culture-based diagnosis	Identification of fungal species.	Sensitivity, Specificity, Accuracy, AUC.
Wang et al., 2023 [21]	Case–control, retrospective study	Total: 485 patients (173 proven IPA, 312 nonfungal pneumonia). Internal training + test: 74 IPA + 74 controls (from 2 hospitals). External validation: 46 IPA + 46 controls (from 1 chest hospital).	Respiratory	Development of an AI-based deep learning diagnostic model (IPA-NET) for early detection of Invasive Pulmonary Aspergillosis (IPA), using chest CT images combined with clinical features.	IPA-NET (transfer learning with 300k CT images), IPA-NET1 (transfer learning with 1.2M ImageNet images), DenseNet121, ResNet50, VGG19, Inception-V3.	Conventional diagnostic methods	Detection of invasive pulmonary aspergillosis (IPA)	Accuracy, sensitivity, specificity
Wei et al., 2023 [22]	Retrospective	234 original images (101 cryptococcosis, 133 talaromycosis), expanded to 1170 images after augmentation	Dermatology	Recognition of cryptococcosis and talaromycosis skin lesions.	VGG19, MobileNet, InceptionV3, Inception ResNetV2, and DenseNet201.	Traditional clinical diagnosis	Classification of fungal infection	Accuracy, sensitivity, and specificity
Xu et al., 2023 [23]	Retrospective	3205 Raman spectra from 123 fungal isolates representing 9 clinical fungal species. External validation set included 14 clinical isolates with 338 spectra.	Non-imaging and molecular AI diagnostic approaches	Application of single-cell Raman spectroscopy (SCRS) combined with machine learning for rapid identification of clinical fungal infections.	Logistic Regression (LR), Linear Discriminant Analysis (LDA), k-Nearest Neighbors (kNN), Support Vector Machine (SVM).	Standard laboratory testing.	Fungal species classification.	Accuracy, sensitivity, and specificity
Zhu et al., 2022 [24]	Retrospective	Training set: 166 OM, 183 Psoriasis, 90 Traumatic onychodystrophy, 188 Normal Test set: 129 OM, 29 Psoriasis, 11 Traumatic onychodystrophy, 39 Normal (Total = 208 images)	Dermatology	Diagnosis of OM was made with direct potassium hydroxide (KOH) examination and culture. Nail clipping or biopsy with periodic acid–Schiff (PAS) staining was performed in all cases of traumatic onychodystrophy and limited cases of OM and nail psoriasis. The final diagnosis was made based on history, physical and mycological testing	Faster R-CNN (3 models) + Ensemble Model Model 1: Normal vs. Nail disorder Model 2: Onychomycosis vs. Other nail disorders Model 3: Detection of 5 dermoscopic patterns (high-specificity predictors) Final output = Ensemble of the three models	KOH microscopy, culture, PAS	Nail disorder detection -Onychomycosis detection	Accuracy, sensitivity, specificity,

Al, artificial intelligence; AK, acanthamoeba keratitis; AUC, area under the receiver-operating characteristic curve; CNN, convolutional neural network; CT, computed tomography; DL, deep learning: DT, decision tree; ENT, Ear, Nose and Throat; FTIR, Fourier-transform infrared; FK, fungal keratitis; IVCM, in vivo confocal microscopy; IPA, invasive pulmonary aspergillosis; kNN, k-Nearest Neighbors; KOH, potassium hydroxide; LR, Logistic Regression; LDA, Linear Discriminant Analysis; NSK, nonspecific keratitis; OM, otomycosis; PPV, positive predictive value; NPV, negative predictive value; PLS-DA, Partial Least Squares–Discriminant Analysis; PCP, Pneumocystis jirovecii pneumonia; PAS, periodic acid–Schiff; RF, random forest; SVM, Support Vector Machine; SCRS, single-cell Raman spectroscopy.

**Table 2:** Classification of diagnostic performance metrics.

Author, (Year), Country	Accuracy (%)	Sensitivity (%)	Specificity (%)
Elkadi et al., 2021 [14]	BaselineM (PLS-DA only): 84.4%	BaselineM (PLS-DA only): 83.3%	BaselineM (PLS-DA only): 85.7%
	AvgSimM (PLS-DA + Oversampling)	AvgSimM (PLS-DA + Oversampling)	AvgSimM (PLS-DA + Oversampling)
	: 91.1%	: 83.3%	: 100%
	BaselineMs (PLS-DA + Autoscaling)	BaselineMs (PLS-DA + Autoscaling)	BaselineMs (PLS-DA + Autoscaling)
	: 93.3%	: 87.5%	: 100%
	AvgSimMs (PLS-DA + Oversampling + Autoscaling)	AvgSimMs (PLS-DA + Oversampling + Autoscaling)	AvgSimMs (PLS-DA + Oversampling + Autoscaling)
	: 93.3%	: 87.5%	: 100%
Essalat et al., 2023 [15]	DenseNet161: 96.93%	DenseNet161: 94.77%	DenseNet161: 97.80%
	DenseNet121: 87.29%	DenseNet121: N/A	DenseNet121: N/A
	ResNet101: 83.59%	ResNet101: N/A	ResNet101: N/A
	ResNet152: 88.01%	ResNet152: N/A	ResNet152: N/A
	VGG19: 90.57%	VGG19: N/A	VGG19: N/A
	VGG13: 92.33%	VGG13: N/A	VGG13: N/A
Kim et al., 2020 [16]	CNN performance:	CNN performance:	CNN performance:
	71.5%	70.2%	72.7%
	Dermoscopy performance:	Dermoscopy performance:	Dermoscopy performance:
	72.8%	72.7%	72.9%
Li et al., 2023 [17]	Random Forest: AUC: 0.907	Random Forest: 80.7%	Random Forest: 83.4%
	XGBoost: AUC: 0.901	XGBoost: 81%	XGBoost: 84.1%
	LightGBM: AUC: 0.888	LightGBM: 80.1%	LightGBM: 82.7%
	Logistic Regression: AUC: 0.855	Logistic Regression: 78.3%	Logistic Regression: 81.3%
Mao et al., 2022 [18]	ResNet101: 78.32%	ResNet101: 73.8%	ResNet101: 86.75%
	SENet101: 87.16%	SENet101: 89.25%	SENet101: 89.82%
	EfficientNetB6: 88.21%	EfficientNetB6: 95.19%	EfficientNetB6: 86.83%
	Ensemble (set classifier): 92.42%	Ensemble (set classifier): 94.65%	Ensemble (set classifier): 95.68%
Soleimani et al., 2023 [19]	Model 1: 99.27%	Model 1: 99.29%	Model 1: 99.19%
	Model 2: 83.99%	Model 2: 84%	Model 2: 84%
	Model 3: 77.5%	Model 3:77.47%	Model 3: 76.58%
Tang et al., 2023 [20]	Fusarium	Fusarium	Fusarium
	DL:	DL:	DL:
	81.7%	79.1%	83.1%
	DT:	DT:	DT:
	70.4%	71.3%	69.9%
	Aspergillus	Aspergillus	Aspergillus
	DL:	DL:	DL:
	75.7%	75.6%	75.9%
	DT:	DT:	DT:
	66.2%	71.1%	62.8%
Wang et al., 2023 [21]	IPA-NET (transfer learning with 300k CT images):	IPA-NET (transfer learning with 300k CT images):	IPA-NET (transfer learning with 300k CT images):
	Internal test:	Internal test:	Internal test:
	96.8%	98%	96%
	External test:	External test:	External test:
	89.7%	88%	91%
	IPA-NET1 (transfer learning with 1.2M	IPA-NET1 (transfer learning with 1.2M	IPA-NET1 (transfer learning with 1.2M
	ImageNet images):	ImageNet images):	ImageNet images):
	94.3%	96%	92%
	DenseNet121:	DenseNet121:	DenseNet121:
	92.9%	92%	94%
	ResNet50:	ResNet50:	ResNet50:
	90.7%	91%	90%
	VGG19:	VGG19:	VGG19:
	90%	91%	89%
	Inception-V3:	Inception-V3:	Inception-V3:
	90.2%	88%	93%
Wei et al., 2023 [22]	VGG19:	VGG19:	VGG19:
	77.04%	79.26%	72.81%
	MobileNet:	MobileNet:	MobileNet:
	77.95%	80.65%	72.81%
	InceptionV3:	InceptionV3:	InceptionV3:
	85.80%	82.49%	92.11%
	InceptionResNetV2:	InceptionResNetV2:	InceptionResNetV2:
	84.89%	85.25%	84.21%
	DenseNet201:	DenseNet201:	DenseNet201:
	87.92%	84.79%	93.86%
Xu et al., 2023 [23]	LDA: 97.5%	LDA: 95%	LDA: 99%
	SVM: 98%	SVM: 97%	SVM: 99%
	kNN: 94.5%	kNN: 92%	kNN: 94.5%
	LR: 93%	LR: 90%	LR: 93%
Zhu et al., 2022 [24]	Nail Disorder Detection (Ensemble Model): 95.7%	Nail Disorder Detection (Ensemble Model): 98.8%	Nail Disorder Detection (Ensemble Model): 82.1%
	Onychomycosis Detection (Ensemble Model): 87.5%	Onychomycosis Detection (Ensemble Model): 93%	Onychomycosis Detection (Ensemble Model): 78.5%

AUC, area under the receiver-operating characteristic curve; DL, deep learning: DT, decision tree; N/A, Not Available.

## Data Availability

Not applicable.

## Supplementary Materials

The following supporting information can be downloaded at: https://www.mdpi.com/article/10.3390/diagnostics16030450/s1, File S1: PRISMA 2020 Checklist. File S2: Risk of Bias Assessment Tool Methodological Index for Nonrandomized Studies (MINORS).
