# Supplementary material for: Advancements in Artificial Intelligence-Based Diagnostic Tools Used to Detect Fungal Infections: A Systematic Review
Source: Diagnostics (Basel). 2026 Feb 1;16(3):450. doi: 10.3390/diagnostics16030450 (PMC12896972; doi:10.3390/diagnostics16030450)
Supplement: Supplementary file 1 [file diagnostics-16-00450-s001.zip › diagnostics-3982233 File S2 diagnostics-3982233-supplementary.pdf]

Risk of Bias Assessment Tool Methodological Index for Nonrandomized Studies (MINORS)

Article	A clearly stated aim	Inclusion of consecutive patients	Prospective collection of data	Endpoints appropriate to the aim of the study	Unbiased assessment of the study endpoint	Follow-up period appropriate to the aim of the study	Loss to follow-up less than 5%	Prospective calculation of the study size	An adequate control group	Contemporary groups	Baseline equivalence of groups	Adequate statistical analyses	Total Score
Elkadi et al., 2021[14]	2	0	2	2	1	2	0	0	2	2	0	1	14
Essalat et al., 2023[15]	2	1	0	2	1	2	0	0	2	1	0	1	12
Kim et al., 2020 [16]	2	2	2	2	1	2	0	0	2	2	0	2	17
Li et al.,2023 [17]	2	0	0	2	0	2	0	0	0	0	0	2	8
Mao et al., 2022 [18]	2	1	0	2	1	2	0	0	2	2	0	1	13
Soleimani et al., 2023 [19]	2	1	0	2	1	2	0	0	1	2	0	1	12
Tang et al., 2023 [20]	2	0	0	2	0	2	0	0	0	0	0	2	8
Wang et al., 2023 [21]	2	1	0	2	1	2	0	0	2	2	1	2	15
Wei et al., 2023 [22]	2	0	0	2	0	2	0	0	0	0	0	2	8
Xu et al., 2023 [23]	2	0	0	2	0	2	0	0	0	0	0	2	8
Zhu et al., 2022 [24]	2	0	0	2	0	2	0	0	0	0	0	2	8
